# Oral nano‐curcumin on gingival inflammation in patients with gingivitis and mild periodontitis

**DOI:** 10.1002/cre2.330

**Published:** 2020-09-21

**Authors:** Meisam Malekzadeh, Seyed Javad Kia, Leila Mashaei, Mahdieh‐Sadat Moosavi

**Affiliations:** ^1^ Dental Sciences Research Center, Department of Periodontics, School of Dentistry Guilan University of Medical Sciences Rasht Iran; ^2^ Dental Sciences Research Center, Department of Oral and Maxillofacial Medicine, School of Dentistry Guilan University of Medical Sciences Rasht Iran; ^3^ Dentist Rasht Iran; ^4^ Dental Research Center, Dental Research Institute, Department of Oral and Maxillofacial Medicine, Faculty of Dentistry Tehran University of Medical Sciences Tehran Iran

**Keywords:** curcumin, gingivitis, nano‐biomedicine, periodontitis

## Abstract

Gingivitis can trigger gingival diseases such as periodontitis. Since the complete removal of microbial plaques by mechanical procedures is not conceivable in some conditions and also chemical mouthwashes have a lot of side effects, finding a new treatment strategy would be useful. In the present study, for the first time, the effects of oral nano‐curcumin on gingival inflammation in patients with gingivitis and mild periodontitis were assessed. Forty eight patients with gingivitis and mild periodontitis participated in this clinical trial. In one group the patients were treated with Sina curcumin capsules 80 mg and the other group received a placebo. Clinical parameters, including modified gingival index, papillary bleeding index, and plaque index were determined on days 0, 7, 14, and 28. There were no significant differences in age, sex, papillary bleeding index (PBI), and modified gingival index (MGI) between the two groups at baseline. There was a dropout of two patients (both from the placebo group). The MGI and PBI have a significantly decreasing trend in both case and control groups and the decreases were severe in the case group. The differences between PBI and MGI in the two groups were significant at 14 and 28 days. The plaque index did not significantly change in either group over the study period. The trend of changes in plaque index was not different between the two groups of the study. In the current study, no side effect was found in the patients. Oral nano‐curcumin has positive effects on the decrease of inflammation and gingival bleeding in patients with gingivitis and mild periodontitis. Nano‐curcumin capsules have a systemic target site with more bioavailability than topical forms.

## 1 INTRODUCTION

Epidemiological studies show that more than 82% of juveniles in the United States have gingivitis and gingival bleeding and a similar or higher prevalence of the disease has been reported in other countries. Gingivitis can trigger gingival diseases such as periodontitis (Farjana, Chandrasekaran, & Gita, 2014).

Periodontal infections can increase the risk of coronary heart diseases, diabetes mellitus, and preterm delivery (Liccardo et al., 2019; Saini, Saini, & Saini, 2010). Gingivitis is the most common form of gingival diseases caused by dental plaque (Farjana et al., 2014).

The critical role of dental plaque in the progression of dental caries, gingival inflammation, and periodontitis has been addressed in the literatures. Therefore, the control and elimination of dental plaque is an essential step in the prevention of periodontal diseases (Farjana et al., 2014).

The control of dental plaque through mechanical procedures is a necessary step in the control of gingivitis and periodontitis. Mechanical removal of gingival plaque is an efficient method for plaque control and gingival inflammation. Because of irregularity and incorrect tooth‐brushing and tooth‐flossing techniques, a large percentage of the population suffer from periodontal diseases (Christie, Claffey, & Renvert, 1998; Nogueira‐Filho, Toledo, & Cury, 2000; Yeturu, Acharya, Urala, & Pentapati, 2016).

Removal of this microbial layer in the advanced stages is doable only by a general dentist, gingival surgeon, or with the aid of special equipment. However, complete elimination of microbial plaque and stimulant agents is not possible solely by employing mechanical procedures (Anuradha et al., 2015; Christie et al., 1998; Nogueira‐Filho et al., 2000; Yeturu et al., 2016).

Chemical approaches, such as mouthwashes, can be used as an adjunct to mechanical microbial plaque control. Although chemical mouthwashes, including chlorhexidine, triclosan, and phenolic agents decrease microbial plaque, they have number of side effects such as allergic reactions and change in tooth color and taste sense (Farjana et al., 2014).

Traditionally, herbal drugs have been used in the treatment of various diseases (Nagpal & Sood, 2013). Turmeric is one of the biocompatible plants that is shown to have a therapeutic effect on recurrent aphthous stomatitis, oral lichen planus, gingivitis, and periodontitis (Deshmukh & Bagewadi, 2014; Farjana et al., 2014; Lv, Chen, Wang, Yao, & Yao, 2018; Nagpal & Sood, 2013). Turmeric is a member of the ginger family and it is extracted from the rhizomes of the *Curcuma longa* Linn plant. Turmeric is a yellow flavored spice that can grow approximately up to a height of 1 meter, has spear‐shaped leaves, and its orange pulp grows in the rhizomes of the plan (Izui et al., 2016; Kunnumakkara et al., 2017; Samal, 2017; Shah, 2017; Zou, Helson, Maitra, Stern, & McNeil, 2013).

The active component of Turmeric is curcuminoid. Ninety percent of curcuminoid is in the form of curcumin (diferuloylmethane) and the remaining of the 10% is in the form of demethoxy curcumin and bis demethoxy curcumin (Farjana et al., 2014).

Curcumin extracted from the *Curcuma longa* Linn plant is a polyphenolic component and generally is known as turmeric. Curcumin exerts its anti‐inflammatory effects by inflammatory pathways regulations and transcription factors such as nuclear factor kappa‐light‐chain‐enhancer of activated B cells (NFKB), activator protein1 (AP‐1), and mitogen‐activated protein kinase (MAP Kinas) (Farjana et al., 2014; Guimarães et al., 2011).

Previous studies showed that the local uses of curcumin gel decreased gingival inflammation and improved the severity of disease. Also, there is evidence that supports curcumin effectively prevents the activation of inflammatory mediators and has therapeutic effects on periodontal diseases (Farjana et al., 2014; Guimaraes‐Stabili et al., 2019).

Curcumin in non‐nano‐formulated products, could stain the teeth and mucosa through local use and have low bioavailability (Jacob, Wu, Zhou, & Wang, 2007; Karabasz et al., 2019; Noorafshan & Ashkani‐Esfahani, 2013).

Regarding the lipophilic nature of curcumin, the oral absorption of curcumin in its usual form (powder, tablet, and capsule) is very low. However, in nano‐curcumin products, all curcumin is trapped in hydrophobic nanomicelles. These spherical shape nanomicelles are ∼10 nm in size and increase the solubility of curcumin in water. After oral consumption, the soft capsules containing curcumin nanomicelles are dissolved in the acidic medium of the stomach in less than 15 minutes. The nanomicelles are stable in the acidic medium of the stomach for at least 6 hours and are transferred to the small intestine without a change in their primary form. In the small intestine, the nanomicelles facilitate the transport of curcumin across the epithelial cells of the small intestine, a barrier against lipophilic substances, and increase the absorption of curcumin when prescribed orally (Muglikar, Patil, Shivswami, & Hegde, 2013; Staff TP, 2004). Curcumin is transferred to other tissues by the bloodstream after absorption in the intestine. Because the inflamed areas have more blood supply (angiogenic response), curcumin can easily reach to the inflamed gingiva tissue (Pober & Sessa, 2015).

Finally, regarding the high prevalence of inflammatory diseases, the side effects of chemical mouthwashes, inflammatory nature of gingivitis and periodontitis, and anti‐inflammatory effects of curcumin, in the present study, the effects of nano‐curcumin capsules on gingival inflammation were investigated in patients with gingivitis and mild periodontitis. It was hypothesized that the indices of inflammation in the before and after treatment in the nano‐curcumin group would be different from those in the placebo group.

## 2 MATERIALS AND METHODS

This double‐blind randomized clinical trial was performed on patients who have been referred to the Periodontics Department. To determine the sample size concerning the repetition of the sizes in the two groups for each individual, the effect of each individual was to be taken into account. The formula for duplicate sizes is used to do this. Considering four replications, a correlation of 0.50, the statistical power of 95%, error level of 0.05, and variance obtained from previous studies equal to 0.09 (Anuradha et al., 2015), the minimum sample size was calculated as 24 patients for each group.

$$n=\frac{2{\left(z_{\frac{\alpha }{2}}+z_{\beta }\right)}^{2}\sigma^{2}\left(1+\left(1-m\right)\rho \right)}{md^{2}}=\frac{2{\left(1.96+1.64\right)}^{2}0.09\left(1+\left(3\right)0.5\right)}{4{\left(0.25\right)}^{2}}=23.33≅24.$$

By taking into consideration the possible loss of some patients, there were 50 patients included in the study. For randomization R software version 3.4.3 was used. The inclusion criteria were as follows: aged between 16–60 years, having at least 20 teeth, a clinical sign of generalized plaque‐induced gingivitis and mild periodontitis (bleeding on probing ≥30%) with CAL 1–2 mm and probing depth ≤ 3, and no history of periodontal treatment in the past 6 months (Trombelli, Farina, Silva, & Tatakis, 2018). Patients who were allergic to turmeric, patients with a history of gallstone and/or biliary obstruction, patients with increased stomach acidity and/or active gastrointestinal ulcer, patients with medication use in the past 3 months, pregnant women, patients with systemic, liver or immunosuppressive diseases, patients with simultaneous use of anticoagulants and antiplatelet, and smokers were excluded from the study. This clinical trial study was approved by the research ethics committee of the university. Registry code in the Primary WHO clinical trial registry center was IRCT 20180416039327N2. Informed consent was obtained from all subjects.

The study participants were randomly classified into two groups as the control and study groups. The demographic data of study subjects including age, sex, medical history, and history of smoking were collected through a prepared questionnaire. All patients were examined on the first day and the plaque index (PI) (Pers et al., 2005), modified gingival index (MGI) (Gomes, Rekhi, Meru, & Efficacy, 2019), and papillary bleeding index (PBI) (Pers et al., 2005) were determined in the subjects. Williams's probe (Hu‐Friedy) was used for examinations. Then, one group was treated with nanomicelles curcumin soft gel capsules (Sina curcumin, provided by ExirNanoSina Company, Tehran, Iran) 80 mg, once per day after breakfast for 4 weeks and the other group received placebo. Since the teeth and mucosa can be stained through local use of curcumin gel, due to the very low solubility of curcumin in hydrophilic solvents and for increasing its bioavailability, we used nano‐curcumin capsules in the present study. The shape, size, and packing of capsules in both groups were identical. So neither the dentist nor the patients were aware of the type of treatment. The rolling technique was demonstrated to all patients and they were asked not to use other mouthwashes and turmeric in food during the study. MGI, PBI, and PI were assessed for all patients on days 0, 7, 14, and 28 (Mishra et al., 2019; Penmetsa, Vivek, Bhupathi, & Sudha, 2019). At the end of the study, dental scaling was done for all patients to remove dental tartar.

Statistical analysis: SPSS version 24.0 (IBM Corp., Armonk, NY) was used to perform statistical analysis. All results were reported as frequency and mean and standard deviation (mean ± *SD*). Repeated measure analysis of variance was used to assess the time‐dependent changes in the MGI, PI, and PBI during the study period in the study groups. To compare the two groups each time, an independent *t*‐test was applied. Also, the posthoc Bonferroni test was used to explore the differences between the different times in each group.

## 3 RESULTS

**FIGURE 1:**
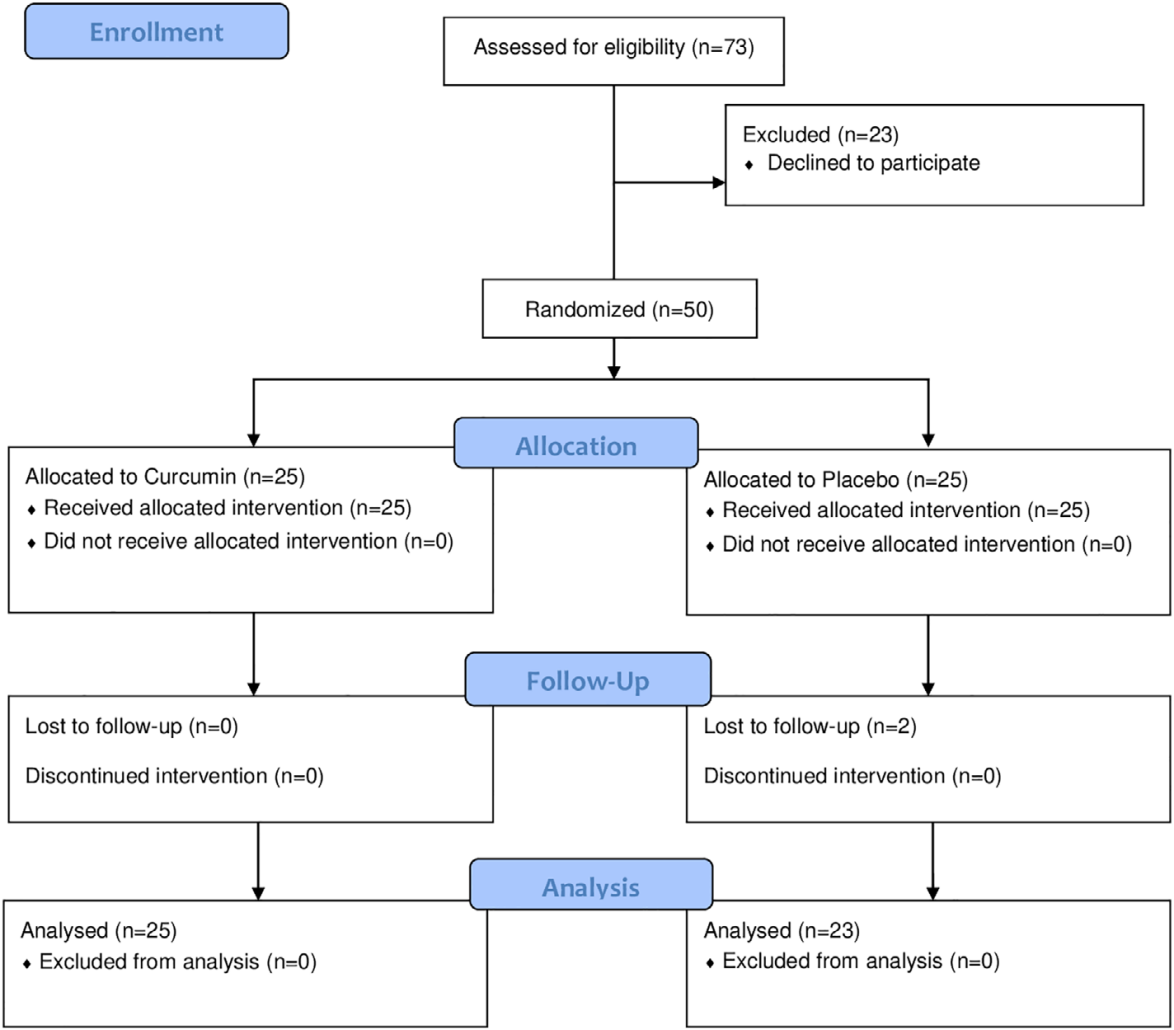
CONSORT flow diagram

Patients included in the study are shown in the CONSORT Flow Diagram (Figure 1). Basic characteristics and clinical indexes at baseline are shown in Table 1. The dropout of two patients (both from the placebo group) was due to the patient's lack of follow‐up. Between the two groups there were no significant differences in age, sex, PBI, and MGI, so intervention and control groups were matched in these parameters. Statistical analysis showed that the papillary bleeding index has a significant decreasing trend over the study period in the case and control groups (*p* < .001). The trend of changes in PBI was different between case and control so that the rate of reduction in PBI in the case group was greater than the control group (*p* < .001) (Table 2). In Table 3 the comparison of PBI between the two groups is shown at each studied time. The differences were significant at 14 and 28 days.

**TABLE 1:** Basic characteristics and clinical indexes at baseline

	Curcumin (*N* = 25)	Placebo (*N* = 23)	*p*‐value
Age (mean ± *SD*) (minimum‐maximum)	30.96 ± 10.10 (16–54)	35.04 ± 11.40 (18–58)	.154
Sex (F/M)	17/8	12/11	.263
Modified gingival index	2.15 ± 0.55	1.95 ± 0.55	.200
Papillary bleeding index	2.10 ± 0.47	1.84 ± 0.58	.089

**TABLE 2:** The results of PBI in case and control groups during 28 days of follow‐up

Time	Group	Mean ± *SD*
Before treatment	Control	1.84 ± 0.58
	Case	2.10 ± 0.47
7th day	Control	1.79 ± 0.57
	Case	1.81 ± 0.47
14th day	Control	1.73 ± 0.56
	Case	1.36 ± 0.43
28th day	Control	1.68 ± 0.57
	Case	1.01 ± 0.38

**TABLE 3:** Comparison of PBI between the two groups

Time	0	7	14	28
Time statistics	1.74	0.11	2.56	4.80
*p*‐value	.089	.915	.014	<.001

Also, statistical analysis showed that the MGI has a significant decreasing trend over the study period in the case and control groups (*p* < .001). The trend of changes in MGI was significantly different between the two groups of the study (*p* < .001) (Table 4). In Table 5 the comparison of MGI between the two groups is shown at each studied time. The differences were significant at 14 and 28 days.

**TABLE 4:** The results of MGI in case and control groups during 28 days of follow‐up

Time	Group	Mean ± *SD*
Before treatment	Control	1.95 ± 0.55
	Case	2.15 ± 0.55
7th day	Control	1.88 ± 0.55
	Case	1.86 ± 0.53
14th day	Control	1.84 ± 0.54
	Case	1.46 ± 0.45
28th day	Control	1.80 ± 0.53
	Case	1.08 ± 0.45

**TABLE 5:** Comparison of MGI between the two groups

Time	0	7	14	28
Time statistics	1.30	0.13	2.67	5.13
*p*‐value	.200	.895	.010	<.001

Regarding the randomization of the patients into two groups, the PI was significant in both groups at baseline. However, the PI did not significantly change in either group over the study period (*p* = .582). The trend of changes in PI was not different between the two groups of the study (*p* = .994).

## 4 DISCUSSION

Gingivitis is a form of periodontal disease that has a high prevalence and usually precedes periodontitis (Farjana et al., 2014). Although the progression of periodontitis is not predictable, its prevention in the earlier stage is still the first step toward preventing periodontitis (Farjana et al., 2014).

In this study, an attempt was made to clarifying the effects of oral nano‐curcumin in gingivitis and mild periodontitis treatment, it was shown that oral nano‐curcumin has positive effects on the decrease of inflammation and gingival bleeding in patients with gingivitis and mild periodontitis. In line with our study findings, the anti‐inflammatory and antioxidant effects of curcumin have been proven in previous studies (Nagpal & Sood, 2013). Also, in the investigation of the impacts of sex and age (>30 years and <30 years) on the effectiveness of curcumin, it was demonstrated that the age and sex have no significant effects on curcumin efficacy.

Given that the study aimed to investigate the effect of adjuvant therapy, the classifying was based on the type of required treatments. Patients who did not require surgical treatment for periodontal disease were entered in the study. Therefore, patients with gingivitis and mild periodontitis were included in the study.

Turmeric includes protein (6.3%), fat (5.1%), minerals (3.5%), curcuminoid (5%), volatile oil (5%), sesquiterepene (alcohol and ketone) and monoterpene (25%). Rhizomes of the *Curcuma longa* Linn plant include arabinose (1%), fructose (12%), glucose (2%), and zinciferous starch grains. The rhizomes also contain curcuminoid, demethoxy curcumin, 5′‐methoxycurcumin, and dihydro curcumin, which have antioxidant properties (Zou et al., 2013).

Curcumin, a main component of turmeric, is inexpensive and available and its antioxidant, anti‐inflammatory, antimicrobial, anti‐pain, anti‐decay, anti‐biofilm, and anti‐cancer effects have been shown in previous studies in the literature. Also, curcumin has protective effects on the liver and kidneys, is an inhibitor of blood coagulation, and prevents myocardial infarction. Turmeric, as an anti‐inflammatory agent, is traditionally used in the management of several diseases. Curcumin improves the performance of the immune system, increases the conservation of the cardiovascular and nervous systems. Also, turmeric extraction prevents the growth of the different strains of pathogenic bacteria and fungi in the mouth and prevents the formation of microbial plaque on the teeth (Corrêa et al., 2017; Nagpal & Sood, 2013; Samal, 2017; Sambhav, Rohit, Ankit Raj, & Garima, 2014; Shah, 2017; Singhla, Tevatia, Chaudhry, Vaish, & Dodwad, 2017; Vaughn et al., 2017).

In the current study, it was concluded that oral nano‐curcumin, due to its anti‐inflammatory effects, can be used as a complementary therapy for gingivitis and mild periodontitis. With respect to our study results, Anuradha et al. investigated the effects of turmeric on patients with localized or generalized chronic periodontitis and showed an anti‐inflammatory effect of turmeric gel. However, they also showed that turmeric gel significantly decreases PI, which was not compatible with our study results (Izui et al., 2016).

Our results were consistent with the study reported by Farjana et al. However, they used the gel form of the *Curcuma longa* plant and had fewer participants compared to our study. Also, the reduction in the PBI in our study was greater than the study reported by Farjana et al, which may be attributed to the use of a different type of turmeric. In other words, the study utilized a topical form of turmeric and therefore its target site is only a local area, while nano‐curcumin capsules have a systemic target site with more bioavailability (Farjana et al., 2014).

Bharat et al. showed that the prevalence of chronic diseases has decreased in people who use curcumin in their daily meals. Although various medications such as steroids, NSAIDs, and chemical mouthwashes are used in the treatment of inflammatory disease, most of them have side effects, especially in long term therapy. Curcumin has a long‐established safety record. In a few studies with high dose levels, some side effects have been reported such as diarrhea, headache, rash, and yellow stool. In the current study, no side effect was found in the patients (Aggarwal & Harikumar, 2009; Hewlings & Kalman, 2017).

Also, Chainani et al. showed that curcumin is a safe component with anti‐inflammatory, anti‐fungi, anti‐viral, and antioxidant effects. In the Chainani et al study similar to the Muglikar et al. study; it was shown that curcumin, besides its mechanical therapeutic strategies, can be used as a complementary therapy to reduce inflammation, but not plaque. Also, Muglikar et al. reported that the anti‐inflammatory effects of curcumin mouthwashes are similar to that of 0.2% chlorhexidine mouthwashes. However, two studies by Gottumukkala et al. and Jalaluddin et al. have reported that 0.2% chlorhexidine mouthwashes have grater effects on clinical parameters such as PI, gingival index, and bleeding on probing index than 1% curcumin mouthwashes. Also, the effects of 1% curcumin mouthwashes on the clinical parameters were much lower than those found in our study. This may be attributed to the low dosage of curcumin and the use of curcumin in the form of mouthwashes. Therefore, it is suggested that a higher dosage of curcumin be used to achieve better results (Chainani‐Wu, 2003; Gottumukkala, Sudarshan, & Mantena, 2014; Jalaluddin et al., 2019; Muglikar et al., 2013). In the current study water solubility, absorption in the gastrointestinal tract, and prolonged plasma half‐life were improved by the formulation of curcumin as nano‐curcumin capsule form.

Since the possibility of Hawthorne‐effect could exist in both intervention and control groups, in the present study, it was not considered as a bias. One of the limitations in our study was the evaluation of solely the clinical outcomes; so, further studies are required to evaluate the therapeutic effects of nano‐curcumin on gingivitis and periodontitis by the means of assessing inflammatory mediators such as TNF‐α and interleukins. Periodontitis is one of the major biofilm‐induced inflammatory diseases. Currently available treatments are not always successful. In the current study the positive effect of curcumin with its anti‐inflammatory effect was approved but, extensive research efforts are needed, and additional anti‐inflammatory approaches should be investigated to improve treatment efficacy(ref). Also, microbiological analysis of biofilm can be one of the goals of future studies in such treatments. It is also recommended that long term follow‐ups be used in future studies to better compare therapeutic effects.

## 5 CONCLUSION

Curcumin is traditionally used for the treatment of several diseases. According to the present study, oral nano‐curcumin, due to its anti‐inflammatory effects, can be used as a complementary therapy for gingivitis and mild periodontitis.

### CLINICAL RELEVANCE

#### The scientific rationale for the study

Gingivitis can trigger gingival diseases such as periodontitis. Complete elimination of microbial plaque and stimulant agents is not possible sloly by mechanical procedures. Nowadays innovated herbal drugs, due to their effectiveness and absence of side effects, are being taken into consideration for the treatment of many disorders.

#### Principal findings

The rate of reduction in PBI and MGI in the case group was significantly greater than the control group.

#### Practical implications

Oral nano‐curcumin, due to its biological effects, can be used as a complementary therapy for gingivitis and mild periodontitis.

## CONFLICTS OF INTEREST

The authors declare no conflicts of interest.

## AUTHOR CONTRIBUTION

Meisam Malekzadeh and Seyed Javad Kia conceived and designed research. Leila Mashaei and Seyed Javad Kia conducted experiments. Mahdieh‐Sadat Moosavi analyzed data and was the main author in writing the manuscript. All authors read and approved the manuscript.

## Supporting information

CONSORT 2010 checklist of information to include when reporting a randomised tria*

## Data Availability

The data that support the findings of this study are available from the corresponding author upon reasonable request.
